# Characteristics of corneal high-order aberrations in adolescents with mild to moderate myopia

**DOI:** 10.1186/s12886-020-01727-z

**Published:** 2020-11-26

**Authors:** Xu Zhang, Jin-Hui Ma, Xin Xi, Lin Guan

**Affiliations:** 1 Baoding Yinghua Eye Hospital, Baoding, 071000 China; 2 Department of endocrinology, Affiliated Hospital of Hebei University, Baoding, 071000 China; 3 Central Laboratory, Affiliated Hospital of Hebei University, No 212. Yuhuadong Road, Lianchi District, Baoding, 071000 China; 4 Department of mathematics, Hebei Agricultural University, Baoding, 071000 China

**Keywords:** Corneal high-order aberration, Myopia, Ocular axial length, Corneal curvatures, Orthokeratology

## Abstract

**Background:**

This study investigated the characteristics of corneal higher-order aberrations (HOAs) of the anterior surface, posterior surface, and total cornea in adolescents with mild to moderate myopia.

**Methods:**

A total of 183 patients with myopia (183 eyes) aged 8 to 18 years were enrolled in this study. The axial length (AL) of the eyes was measured by an IOL-Master, and corneal curvatures (K-values) and HOAs were measured by a Pentacam anterior segment diagnostic analyzer.

**Results:**

Results of this study showed that the anterior, posterior and total corneal horizontal coma Z_3_^1^ were − 0.1249 ± 0.105 μm, 0.0009 ± 0.001 μm, and − 0.1331 ± 0.116 μm, respectively; the anterior, posterior and total corneal vertical coma Z_3_^− 1^ were − 0.0212 ± 0.164 μm, 0.0003 ± 0.043 μm, and − 0.0216 ± 0.168 μm, respectively; and spherical aberration (SA) Z_4_^0^ values were 0.2244 ± 0.091 μm, 0.1437 ± 0.029 um, and 0.1889 ± 0.090 μm, respectively. Total corneal Z_3_^1^ was statistically correlated with posterior corneal astigmatism (K_2_b − K_1_b) (*p* = 0.038). Total corneal Z_3_^− 1^ was correlated with anterior corneal astigmatism (K_2_f − K_1_f) (*p* = 0.027). Anterior, posterior, and total corneal Z_4_^0^ were correlated with anterior and posterior corneal curvature (K_1_f, K_2_f, K_1_b, K_2_b) (*p* = 0.001). Posterior corneal Z_4_^0^b was also significantly correlated with AL.

**Conclusions:**

In adolescents with mild to moderate myopia, the posterior corneal surface shape may play a compensatory role in the balance of corneal aberrations, and the posterior corneal SA tended to become less negative as the AL increased. The corneal coma may also play a compensatory role in posterior corneal surface astigmatism, which was valuable for the treatment for improving visual quality. This conclusion still needs to be verified.

## Background

The prevalence of myopia is high in East Asia, and approximately one in six of the world’s population is myopic [1, 2]. The prevention and control of adolescent myopia are of global importance because of its high burden on vision health. Some scholars believe that the decline in retinal imaging quality caused by high-order aberrations (HOAs) may be a factor that influences the development of myopia in children [3–7]. In a study conducted by Zhang et al., HOA without spherical aberration (SA) was correlated with the progression of myopia [4], and it was predicted that HOA is a risk factor for myopia progression. Animal studies showed that a form of deprivation or lens-induced blur can induce myopia by degrading the quality of the retinal image [5]. Wang et al. [6] suggested that HOAs may be a cause of accommodative anomalies, while Kirwan et al. [7] found that myopic eyes have more HOAs than emmetropic eyes. However, Cheng et al. [8] found no correlation between refractive error diopter and either SA or HOAs. Hiraoka et al. [9] found significant increases in corneal and total ocular HOAs after orthokeratology.

Most of the previous studies of HOAs only focus on myopia in adults. The aberrations of human eyes mainly come from corneal aberrations, which are closely related to the visual quality of the human eyes [10, 11]. Therefore, we conducted this study to investigate the characteristics of corneal HOAs of the anterior surface, posterior surface and total cornea in adolescents with mild to moderate myopic eyes.

## Methods

### Patients

In this study, adolescent myopia patients in Baoding Yinghua Eye Hospital, Baoding City, Hebei Province, China, were recruited between January 2016 and May 2018. This research adhered to the principles highlighted in the Declaration of Helsinki and was approved by the Institutional Review Board of Clinical College of Ophthalmology in Tianjin Medical University. Informed consent was obtained from each participant.

### The inclusion and exclusion criteria

The inclusion criteria were: (1) patients with myopic eyes and best corrected visual acuity above 1.0 without other ocular disease; (2) patients aged between 8 and 18 years; (3) intraocular pressure (IOP) < 21 mmHg; (4) in order to avoid the correlation of binocular growth and the influence of aberrations directionality, all the patients were enrolled in the right eye group.

The exclusion criteria were: (1) patients with a congenital cataract, corneal disease, uveitis, fundus disease, and other eye diseases; (2) patients who had undergone previous intraocular surgery; (3) patients aged less than 8 or more than 18 years; (4) intraocular pressure (IOP) ≥21 mmHg.

### Ocular examinations

The diopter was determined utilizing the cyclopentanone mydriasis and atropine amide mydriasis assays for children aged ≤10 and > 10 years, respectively. The axial length (AL) measurements were taken five times using an intraocular lens biometric instrument (IOL-Master 2.0, Zeiss, Germany), and the values were averaged. Moreover, corneal curvature and aberrations were measured with the patient in the sitting position, with the patients’ natural pupil size, and in a dark environment. An anterior segment analyzer (Pentacam 70,700, Oculus, Germany) was utilized. The Pentacam anterior segment analysis system provides a 360° uniform rotation scanning technique based on the Scheimpflug principle, which is a reliable technique for measuring anterior, posterior, and total corneal aberrations.

After recording the patients’ information, the researchers instructed them to place their lower jaw on the lower jaw rest and their forehead against the forehead rest. The patients’ eyes were then kept wide open, and the patient was instructed to look at the flashing blue lights. The inspector utilized the operating rod according to the screen prompt direction focus. K1 referred to minimum corneal curvature, and K2 referred to maximum corneal curvature. After adjusting the alignment, 25 frames of the Scheimpflug images were utilized to reconstruct the three-dimensional structure of the anterior segment and measure the curvature of the anterior (K_1_f and K_2_f) and posterior (K_1_b and K_2_b) surfaces of the cornea. Zernike was utilized for analyzing the anterior corneal surface (Z_3_^1^f, Z_3_^− 1^f, and Z_4_^0^f), the posterior corneal surface (Z_3_^1^b, Z_3_^− 1^b, and Z_4_^0^b), the and total cornea (Z_3_^1^, Z_3_^−1^, and Z_4_^0^) within the 6-mm diameter range centered on the corneal vertex. Only cases where the quality parameters of the examination were shown as “OK” were selected, which indicated the repeatability and reproducibility of the measurements that could be reproduced for a clinical diagnosis. The abovementioned inspections were performed by the same skilled technician.

### Statistical analysis

We utilized the software program SPSS 22.0 (IBM, Chicago, IL, USA) to conduct the statistical analysis. Continuous variables were expressed as mean ± SD, and discontinuous variables were expressed as a percentage (%). For multiple comparisons, each value was compared by one-way ANOVA following Dunnett’s test when each datum conformed to normal distribution, while the non-normally-distributed continuous data were compared using non-parametric tests. The correlation between the corneal HOAs (Z_3_^1^, Z_3_^− 1^, and Z_4_^0^) on the anterior corneal surfaces, posterior corneal surfaces, total cornea and the eye parameter (AL, anterior and posterior corneal surface curvature) were analyzed utilizing a Pearson correlation analysis. A value of *p* < 0.05 was considered statistically significant, and p was the conducted Bonferroni correction (P/N).

## Results

### Patient characteristics

A total of 183 patients (183 right eyes) were included in this study. The patients’ age ranged from 8 to 18 years, and the average age was 11.8 ± 2.4 years. Among the 183 participants, there were 75 males (41%) and 108 females (59%). The specific baseline characteristics are shown in Table 1.

**Table 1:** Characteristics of the Participants

Parameter	Mean ± SD	Range
Age (years)	11.8 ± 2.4	8 ~ 18
SE (D)	−3.47 ± 0.09	−1.00 ~ −6.00
AL (mm)	24.94 ± 0.88	23.06 ~ 27.52
K_1_f (D)	42.56 ± 0.07	39.20 ~ 48.10
K_2_f (D)	43.73 ± 0.08	39.80 ~ 49.50
K_1_b (D)	−6.15 ± 0.01	−5.60 ~ −6.90
K_2_b (D)	−6.51 ± 0.01	−5.90 ~ −7.80

Note: *SE* Equivalent spherical, *AL* Axial length, *K*_*1*_*f* the K_1_ value of the anterior corneal curvature, *K*_*2*_*f* the K_2_ value of the anterior corneal curvature, *K*_*1*_*b* the K_1_ value of the posterior corneal curvature, *K*_*2*_*b* the K_2_ value of the posterior corneal curvature

### The correlations between corneal HOAs (coma aberration, SA) and ocular parameter (AL, astigmatism, and corneal curvature)

**Fig. 1:**
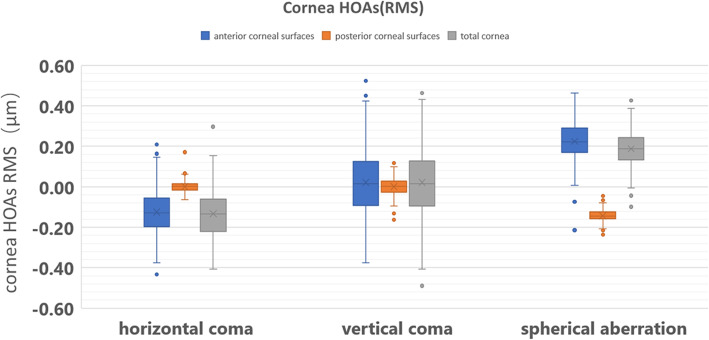
HOAs (μm) of the anterior and posterior corneal surface and the total cornea at 6 mm

The mean ± SD and distribution range of HOAs are shown in Table 2. Figure 1 shows the HOAs of the anterior and posterior corneal surface and the total cornea at 6 mm.

**Table 2:** Mean ± SD of corneal spherical aberration and coma in 183 eyes

	Mean ± SD	Range
Z_3_^1^f	−0.0218 ± 0.0071	− 0.43 ~ 0.47
Z_3_^1^b	0.0089 ± 0.0014	−0.06 ~ 0.17
Z_3_^1^	−0.0142 ± 0.0082	−0.41 ~ 0.91
Z_3_^−1^ f	−0.0109 ± 0.0082	− 0.38 ~ 0.63
Z_3_^− 1^ b	0.0093 ± 0.0021	− 0.21 ~ 0.12
Z_3_^− 1^	− 0.0019 ± 0.0083	− 0.49 ~ 0.07
Z_4_^0^ f	0.2281 ± 0.0042	− 0.21 ~ 0.46
Z_4_^0^ b	−0.1485 ± 0.0017	−0.28 ~ 0.18
Z_4_^0^	0.1843 ± 0.0044	−0.10 ~ 0.45

Note: Z_3_^1^f, horizontal coma aberration of the anterior corneal surface; Z_3_^1^b, horizontal coma aberration of the posterior corneal surface; Z_3_^1^, horizontal coma aberration of the total cornea; Z_3_^−1^f, vertical coma aberration of the anterior corneal surface; Z_3_^− 1^b, vertical coma aberration of the posterior corneal surface; Z_3_^− 1^, vertical coma aberration of the total cornea; Z_4_^0^f, spherical aberration of the anterior corneal surface; Z_4_^0^b, spherical aberration of the posterior corneal surface; Z_4_^0^, spherical aberration of the total cornea; Mean ± SD, Mean ± Standard Deviation (μm); Range, min to max (μm)

**Fig. 2:**
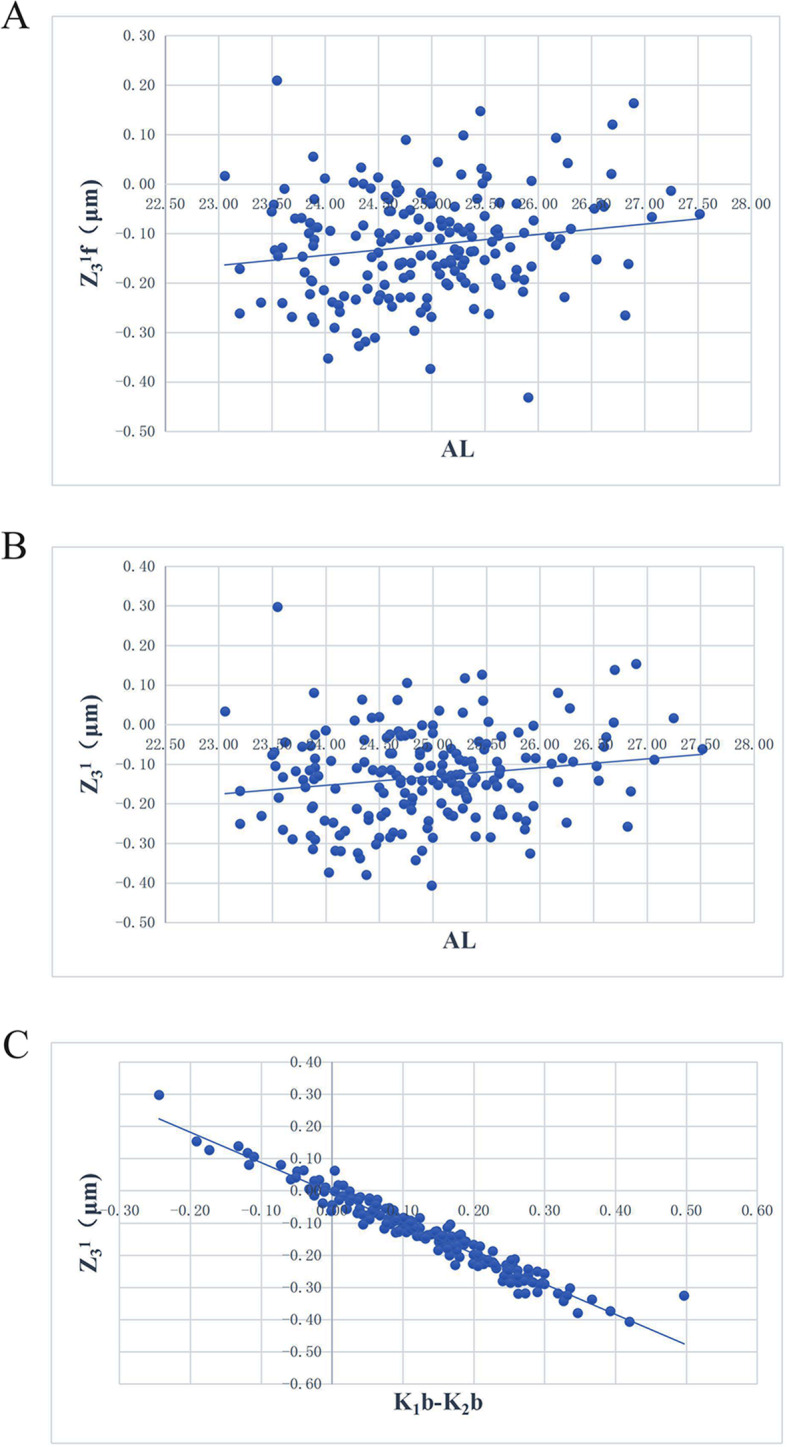
The results of the analysis of the correlation between corneal horizontal coma aberration and ocular parameter. **a**: Scatter diagram of the correlation between horizontal coma of the anterior corneal surface and ocular axis length. **b**: Scatter diagram of the correlation between horizontal coma of the total cornea and ocular axis length. **c**: Scatter diagram of the correlation between horizontal coma of the total cornea and K_1_b − K_2_b

The results of the correlations between corneal horizontal coma aberration and ocular parameter showed that total corneal horizontal coma Z_3_^1^ was significantly correlated with AL and posterior corneal astigmatism (K_2_b-K_1_b) (*r* = 0.171, 0.154, *p* = 0.020, 0.038). Anterior corneal horizontal coma Z_3_^1^f was also significantly correlated with AL (*r* = 0.176, *p* = 0.017). Posterior corneal horizontal coma Z_3_^1^b was significantly correlated with K_2_b (K_2_b − K_1_b) (*r* = − 0.145, 0.188, *p* = 0.050, 0.011) (Table 3, Fig. 2).

**Table 3:** The correlation analysis results of corneal horizontal coma aberration with ocular parameter

Ocular parameter	Z_**3**_^**1**^ f		Z_**3**_^**1**^ b		Z_**3**_^**1**^	
	***r***	***P***	***r***	***P***	***r***	***P***
**K**_**1**_**f**	−0.059	0.427	0.085	0.252	−0.040	0.606
**K**_**2**_**f**	−0.064	0.390	0.094	0.207	−0.043	0.562
**K**_**1**_**b**	0.034	0.644	−0.028	0.709	0.033	0.655
**K**_**2**_**b**	−0.041	0.584	−0.134	0.070	−0.065	0.385
**AL**	0.176	0.018	−0.003	0.973	0.169	0.022
**astigmatism**	0.019	0.802	0.085	0.254	0.042	0.574
**K**_**2**_**f -K**_**1**_**f**	−0.028	0.709	0.037	0.621	−0.022	0.765
**K**_**2**_**b -K**_**1**_**b**	0.117	0.114	0.182	0.013	0.155	0.036

Note: *AL* Axial length, *K*_*1*_*f* the K_1_ value of the anterior corneal curvature, *K*_*2*_*f* the K_2_ value of the anterior corneal curvature, *K*_*1*_*b* the K_1_ value of the posterior corneal curvature, *K*_*2*_*b* the K_2_ value of the posterior corneal curvature

Pearson correlation analysis was used

**Fig. 3:**
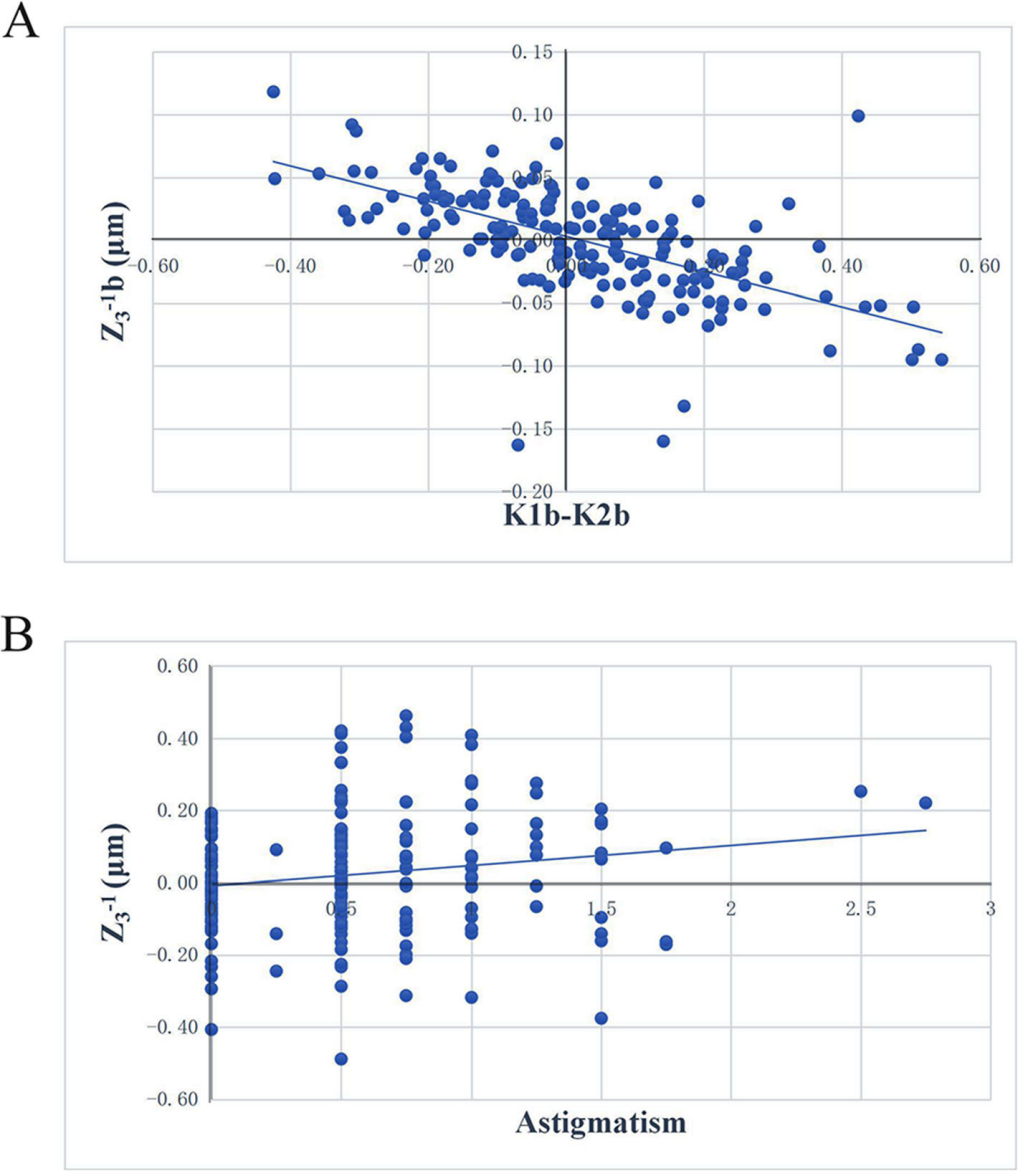
The results of the analysis of the correlation between corneal vertical coma aberration and ocular parameter. **a**: Scatter diagram of the correlation between vertical coma of the posterior corneal surface and K_1_b − K_2_b. **b**: Scatter diagram of the correlation between vertical coma of the total cornea and astigmatism

The results of the correlations between corneal vertical coma aberration and ocular parameter showed that total corneal vertical coma Z_3_^− 1^ was significantly correlated with K_1_b and anterior corneal astigmatism (K_2_f − K_1_f) (r = − 0.151, 0.163, *p* = 0.041, 0.027). Anterior corneal vertical coma Z_3_^−1^f was significantly correlated with K_1_b and astigmatism (*r* = − 0.167, 0.168, *p* = 0.024, 0.023). Posterior corneal vertical coma Z_3_–^1^b was significantly correlated with posterior corneal astigmatism (K_2_b − K_1_b) (*r* = 0.158, *p* = 0.032) (Table 4, Fig. 3).

**Table 4:** The correlation analysis results of corneal vertical coma aberration with ocular parameter

Ocular parameter	Z_**3**_^**−1**^ f		Z_**3**_^**−1**^ b		Z_**3**_^**−1**^	
	***r***	***P***	***r***	***P***	***r***	***P***
**K**_**1**_**f**	0.081	0.275	−0.109	0.144	0.048	0.520
**K**_**2**_**f**	0.124	0.095	−0.103	0.164	0.106	0.153
**K**_**1**_**b**	−0.168	0.023	0.104	0.161	−0.151	0.042
**K**_**2**_**b**	−0.119	0.108	−0.004	0.955	−0.127	0.087
**AL**	0.016	0.835	0.017	0.816	0.077	0.302
**astigmatism**	0.143	0.054	0.042	0.576	0.170	0.022
**K**_**2**_**f -K**_**1**_**f**	0.132	0.075	−0.014	0.854	0.165	0.026
**K**_**2**_**b -K**_**1**_**b**	−0.046	0.538	0.158	0.033	−0.008	0.911

Note: *AL* Axial length, *K*_*1*_*f* the K_1_ value of the anterior corneal curvature, *K*_*2*_*f* the K_2_ value of the anterior corneal curvature, *K*_*1*_*b* the K_1_ value of the posterior corneal curvature, *K*_*2*_*b* the K_2_ value of the posterior corneal curvature

Pearson correlation analysis was used

**Fig. 4:**
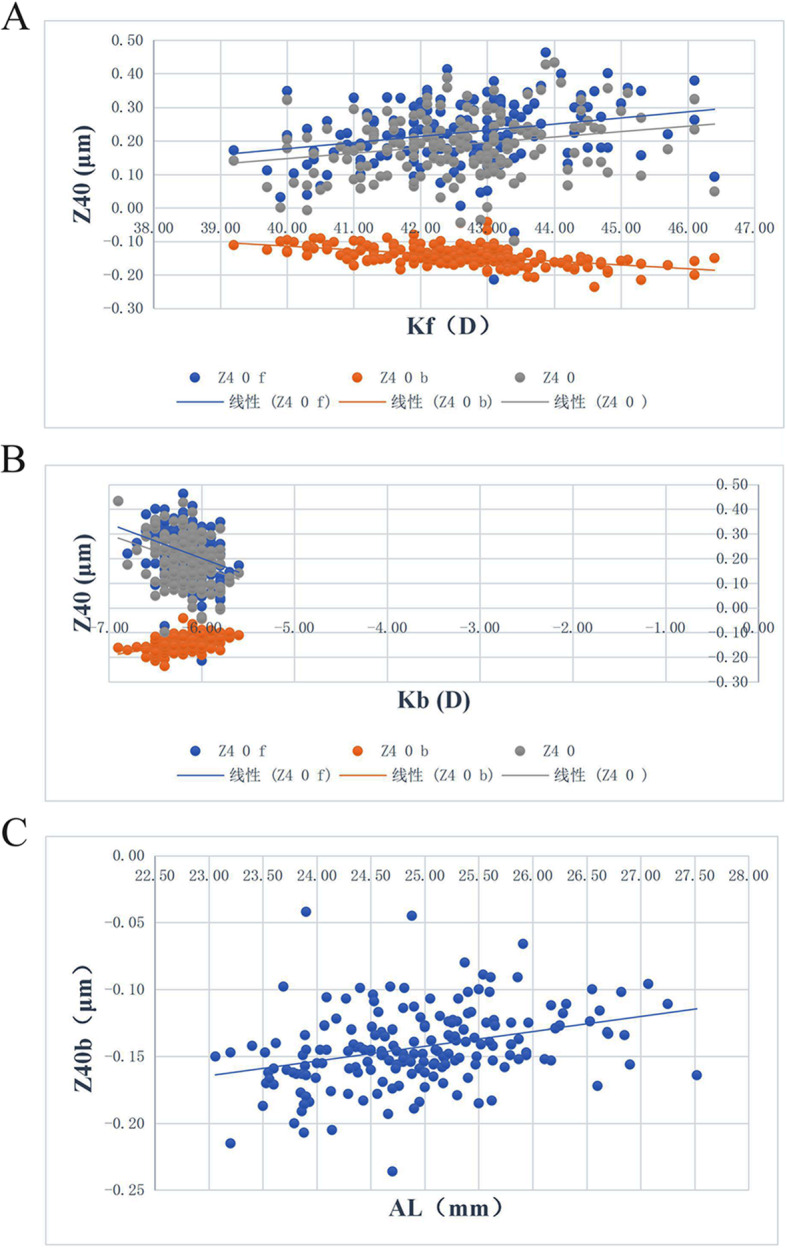
The results of the analysis of the correlation between corneal spherical aberration and ocular parameter. **a**: Scatter diagram of the correlation between spherical aberration of the anterior and posterior corneal surface and total cornea with anterior cornea curvature Kf. **b**: Scatter diagram of the correlation between spherical aberration of the anterior and posterior corneal surface and total cornea with posterior cornea curvature Kb. **c**: Scatter diagram of the correlation between spherical aberration of the posterior corneal surface and ocular axis length

The results of the correlations between corneal SA and ocular parameters showed that total corneal SA Z_4_^0^ was significantly correlated with anterior and posterior corneal curvature (K_1_f, K_2_f, K_1_b, K_2_b) (*r* = 0.234, 0.246, − 0.308, − 0.284, *p* < 0.05). Anterior corneal SA Z_4_^0^f was significantly correlated with anterior and posterior corneal curvature (K_1_f, K_2_f, K_1_b, K_2_b) (*r* = 0.260, 0.249, − 0.331, − 0.242, *p* < 0.05). Posterior corneal SA Z_4_^0^b was significantly correlated with anterior and posterior corneal curvature (K_1_f, K_2_f, K_1_b, K_2_b), AL, and posterior corneal astigmatism (K_2_b − K_1_b) (r = − 0.521, − 0.460, 0.464, 0.297, 0.344, 0.180, p < 0.05) (Table 5, Fig. 4).

**Table 5:** The correlation analysis results of corneal spherical aberration with ocular parameter

Ocular parameter	Z_**4**_^**0**^ f		Z_**4**_^**0**^ b		Z_**4**_^**0**^	
	***r***	***P***	***r***	***P***	***r***	***P***
**K**_**1**_**f**	0.262	< 0.001	−0.514	< 0.001	0.234	0.001
**K**_**2**_**f**	0.251	0.001	−0.452	< 0.001	0.247	0.001
**K**_**1**_**b**	−0.332	< 0.001	0.456	< 0.001	−0.309	< 0.001
**K**_**2**_**b**	−0.245	0.001	0.289	< 0.001	−0.286	< 0.001
**AL**	−0.116	0.117	0.335	< 0.001	−0.108	0.146
**astigmatism**	0.121	0.104	−0.086	0.244	0.107	0.149
**K**_**2**_**f -K**_**1**_**f**	0.038	0.611	0.030	0.684	0.094	0.204
**K**_**2**_**b -K**_**1**_**b**	−0.076	0.309	0.182	0.014	0.027	0.717

Note: *AL* Axial length, *K*_*1*_*f* the K_1_ value of the anterior corneal curvature, *K*_*2*_*f* the K_2_ value of the anterior corneal curvature, *K*_*1*_*b* the K_1_ value of the posterior corneal curvature, *K*_*2*_*b* the K_2_ value of the posterior corneal curvature

Pearson correlation analysis was used

### The correlations between corneal HOAs and age

Posterior corneal horizontal coma Z_3_^1^b, total corneal SA Z_4_^0^ and anterior corneal SA Z_4_^0^f were significantly correlated with age (*r* = 0.272, 0.199, 0.191, *p* < 0.05) (Table 6).

**Table 6:** The correlation analysis results of corneal high order aberration with age, diopter

Aberration	Age		Diopter	
	***r***	***P***	***r***	***P***
**Z**_**3**_^**1**^**f**	0.050	0.505	0.046	0.536
**Z**_**3**_^**1**^**b**	0.272	< 0.001	0.048	0.521
**Z**_**3**_^**1**^	0.107	0.151	0.050	0.505
**Z**_**3**_^**−1**^**f**	0.064	0.389	0.127	0.086
**Z**_**3**_^**−1**^**b**	0.073	0.326	−0.027	0.719
**Z**_**3**_^**−1**^	0.103	0.167	0.188	0.011
**Z**_**4**_^**0**^**f**	0.199	0.007	0.155	0.036
**Z**_**4**_^**0**^**b**	−0.032	0.666	0.047	0.529
**Z**_**4**_^**0**^	0.191	0.010	0.136	0.067

Note: Z_3_^1^f, horizontal coma aberration of the anterior corneal surface; Z_3_^1^b, horizontal coma aberration of the posterior corneal surface; Z_3_^1^, horizontal coma aberration of the total cornea; Z_3_^−1^f, vertical coma aberration of the anterior corneal surface; Z_3_^− 1^b, vertical coma aberration of the posterior corneal surface; Z_3_^− 1^, vertical coma aberration of the total cornea; Z_4_^0^f, spherical aberration of the anterior corneal surface; Z_4_^0^b, spherical aberration of the posterior corneal surface; Z_4_^0^, spherical aberration of the total cornea

Pearson correlation analysis was used

## Discussion

The outcomes of this study showed that total corneal Z_3_^1^ was statistically correlated with posterior corneal astigmatism (K_2_b − K_1_b), and that total corneal Z_3_–^1^ was correlated with anterior corneal astigmatism (K_2_f − K_1_f). Anterior, posterior, and total corneal Z_4_^0^ were correlated with anterior and posterior corneal curvature (K_1_f, K_2_f, K_1_b, K_2_b). Posterior corneal Z_4_^0^b was also significantly correlated with AL.

To the best of our knowledge, there are few studies on the characteristics of corneal HOAs (the anterior surface, posterior surface and total cornea) in adolescents with mild to moderate myopic eyes. Furthermore, the potential relationship between the ocular parameters (AL and anterior and posterior curvature of the cornea) and corneal HOA was examined, which may provide a reference and direction for an individualized approach for the prevention and control of myopia. Further studies are needed to investigate the change in the aberrations in adolescents with myopia.

In this study, we analyzed the characteristics of corneal HOAs in adolescents with mild to moderate myopic eyes. Previously, SA had been considered as an important factor affecting the quality of vision in HOAs [12, 13]. In our study, however, we observed significantly higher SA of the anterior corneal surface than of the total cornea, which suggests that the posterior corneal surface plays a compensatory role in the balance of corneal aberrations in adolescents with mild to moderate myopic eyes. This result was similar to that of Wu et al. [14], who found that the anterior corneal aberrations are similar to the total corneal aberrations, and that the total corneal aberrations are mainly determined by the anterior surface in adults. However, Sicam et al. [15] reported that the posterior corneal surface had a significant effect on the SA of the cornea in adults. Unlike previous studies, our research focused on the corneal aberration of adolescents with mild to moderate myopic eyes. The higher levels of negative SA would produce relative peripheral hyperopic defocus and provided an optical cue for myopia progression [16]. Positive SA was associated with less myopic shift [12], and the coma aberrations found in our study were similar to those found in previous studies [12, 14]. The corneal change induced by the eyelid pressure on the superior side can influence the vertical coma [17].

In this study, the relationship between AL and corneal curvature with corneal SA was analyzed. Anterior, posterior, and total corneal SA were correlated with corneal curvature of the anterior and posterior corneal surface. Since the cornea is flat, the anterior and total corneal SA tend to become less positive or more negative, and the posterior corneal surface SA tends to become less negative. Thus, corneal curvature plays an important role in improving visual quality. The curvature radius of the anterior and posterior surface of the normal cornea from the vertex to the periphery is not the same, there are dissimilarities in its variation, and it has an aspheric surface. Although there was no statistically significant correlation between total corneal SA and ocular AL (*r* = − 0.106, *p* = 0.154), posterior corneal SA was significantly positively correlated with ocular AL (*r* = 0.344, *p* < 0.001), which means that as AL increased, the posterior corneal SA tended to become less negative. In a study of adults, Shimozono et al. [18] found that AL of the eye was significantly negatively correlated with corneal SA (*r* = − 0.135, − 0.201, *p* < 0.05). Furthermore, Nambe et al. [19] and Hidaka et al. [20] confirmed the relationship between age and HOAs. As the patients’ age increases, some HOAs will vary and the AL in adolescent patients will grow, and therefore the relation between ocular AL and corneal SA may vary. The posterior corneal surface compensates for the SA of most anterior corneal surfaces, which reduces the total corneal SA. As the ocular AL grows, the compensatory effect may be changed.

Finally, we analyzed the relation between the corneal coma and the ocular parameter. Posterior and total corneal horizontal coma and posterior corneal vertical coma were correlated with posterior corneal astigmatism (K_2_b − K_1_b) (*p* < 0.05). As the posterior corneal astigmatism (K_2_b − K_1_b) increased, the posterior total corneal horizontal coma and posterior corneal vertical coma tended to become more positive or less negative. Total corneal vertical coma was significantly correlated with anterior corneal astigmatism (K_2_f − K_1_f) (*p* < 0.05). As the anterior corneal astigmatism (K_2_f − K_1_f) increased, the posterior and total corneal vertical coma tended to become more positive or less negative. Anterior vertical coma was positively correlated with total astigmatism. As total astigmatism increased, the anterior corneal vertical coma tended to become more positive. Koch et al. [21] reported that neglecting astigmatism on the posterior surface of the cornea in adults would affect the evaluation of astigmatism on the whole cornea. The posterior surface of the cornea has important optical significance; it may play an important role in optical compensation and optimization of visual quality. However, this theory needs to be explored further in future studies.

In this study, some HOAs were significantly correlated with age, including posterior corneal horizontal coma Z_3_^1^b, total corneal SA Z_4_^0^, and anterior corneal SA Z_4_^0^f. In a previous study, Wang et al. [22] showed that aberration changes with age in adults, but this needs to be verified in studies with longer follow-up durations. There was no significant correlation between corneal HOAs and diopter of myopia, which may be different to that found in adults. There is some controversy regarding the relationship between human eye HOAs and myopia diopter. Some studies [23, 24] showed more positive HOAs in higher diopter myopia, whereas other studies [25, 26] showed less positive HOAs in higher diopter myopia. Moreover, other research studies [8, 25] showed no relationship between HOAs and myopia diopter. Our study focused on cornea HOAs and mild to moderate myopic eyes in adolescents. HOAs reflected the precision of the human eye optical system. HOAs have no obvious correlation with diopter of myopia but are directly related to corneal curvature and AL. The magnitude of aberration is mainly due to the combination of optical elements in the ocular itself. However, many aberrations are compensated [27], and therefore not only should the relationship between aberration and diopter be analyzed, but also the corneal curvature and AL should be considered.

However, there were some limitations in this study. First, this study investigated the characteristics of corneal HOAs of the anterior surface, posterior surface and total cornea in adolescents with mild to moderate myopic eyes. The characteristics of corneal HOAs of the anterior surface, posterior surface and total cornea in other populations are unknown and should be studied further in the future. Second, this study was only a single-center trial and the sample size was limited. A multiple-center trial with a large sample size is still needed to explore in the future.

## Conclusion

In adolescents with mild to moderate myopic eyes, the posterior corneal surface shape plays a compensatory role in the balance of corneal aberrations, and the posterior corneal SA tended to become less negative as the axial length increased. The corneal coma also plays a compensatory role in posterior corneal surface astigmatism, which was valuable for the treatment for improving visual quality. This conclusion still needs to be verified.

## Data Availability

The datasets generated and/or analysed during the current study are not publicly available due to the lack of an online platform but are available from the corresponding author on reasonable request.

## Abbreviations

HOAs: Higher-order aberrations
AL: Axial length
SA: Spherical aberration
